# The COVID-19 pandemic and vaccination abandonment in children: spatial heterogeneity maps

**DOI:** 10.1590/1518-8345.6132.3642

**Published:** 2022-10-07

**Authors:** Rayssa Nogueira Rodrigues, Gabriela Lourença Martins do Nascimento, Luiz Henrique Arroyo, Ricardo Alexandre Arcêncio, Valéria Conceição de Oliveira, Eliete Albano de Azevedo Guimarães

**Affiliations:** 1 Universidade Federal de Viçosa, Departamento de Medicina e Enfermagem, Viçosa, MG, Brazil.; 2 Universidade Federal de São João del Rei, Campus Centro-Oeste Dona Lindu, Divinópolis, MG, Brazil.; 3 Ministério da Saúde, Secretaria de Vigilância em Saúde, Brasília, DF, Brazil.; 4 Universidade de São Paulo, Escola de Enfermagem de Ribeirão Preto, Centro Colaborador da OPAS/OMS para o Desenvolvimento da Pesquisa em Enfermagem, Ribeirão Preto, SP, Brazil.

**Keywords:** Immunization, Public Health, Child, COVID-19, Spatial Analysis, Ecological Studies, Imunização, Saúde Pública, Criança, COVID-19, Análise Espacial, Estudos Ecológicos, Inmunización, Salud Pública, Niño, COVID-19, Análisis Espacial, Estudios Ecológicos

## Abstract

**Objective:**

to identify spatial clusters corresponding to abandonment of routine vaccines in children.

**Method:**

an ecological study, according to data from the 853 municipalities of a Brazilian state. The records analyzed were those of the multidose pentavalent, pneumococcal 10-valent, inactivated poliomyelitis and oral human rotavirus vaccines of 781,489 children aged less than one year old. The spatial scan statistics was used to identify spatial clusters and assess the relative risk based on the vaccination abandonment indicator.

**Results:**

the spatial scan statistics detected the presence of statistically significant clusters for abandonment regarding the four vaccines in all the years analyzed. However, the highest number of clusters with high relative risk estimates was identified in 2020. The Vale do Aço and West, North and West, and Southwest regions stand out for the pentavalent, poliomyelitis and rotavirus vaccines, respectively.

**Conclusion:**

in an attempt to mitigate the devastating impact of the COVID-19 pandemic, the immunization program experienced setbacks. The presence of clusters points to the need to implement integrated strategies that may involve different sectors for an active search for children and prevent outbreaks of vaccine-preventable diseases in the near future.

## Resumo

**Objetivo:**

identificar aglomerados espaciais de abandono de vacinas de rotina em crianças.

**Método:**

estudo ecológico, segundo dados dos 853 municípios de um Estado brasileiro. Foram analisados registros das vacinas multidoses pentavalente, pneumocócica 10-valente, vacina inativada contra a poliomielite e vacina oral de rotavírus humano de 781.489 crianças menores de um ano de idade. A estatística *scan* espacial foi utilizada para identificar agrupamentos espaciais e medir o risco relativo a partir do indicador de abandono de vacinas.

**Resultados:**

a estatística *scan* espacial detectou a presença de aglomerados estatisticamente significativos para o abandono das quatro vacinas em todos os anos analisados. No entanto, o maior número de aglomerados com elevadas estimativas dos riscos relativos foi identificado no ano de 2020. Destaca-se as macrorregiões do Vale do Aço e Oeste; Norte e Oeste; e Sudeste para as vacinas pentavalente, poliomielite e rotavírus, respectivamente.

**Conclusão:**

na tentativa de mitigar o impacto devastador da pandemia de COVID-19, o programa de imunização retrocedeu. A presença de aglomerados aponta a necessidade de implementar estratégias integradas que possam envolver diferentes setores para a busca ativa de crianças e evitar surtos de doenças imunopreveníveis no futuro próximo.

## Resumen

**Objetivo:**

identificar grupos espaciales que abandonaron la vacunación de rutina de los niños.

**Método:**

estudio ecológico, basado en los datos de 853 municipios de un Estado brasileño. Se analizaron los registros de vacunas multidosis pentavalente, antineumocócica 10-valente y antipoliomielítica inactivada y vacuna oral contra el rotavirus humano de 781.489 niños menores de un año de edad. Se utilizó la estadística *scan* espacial para identificar agrupaciones espaciales y medir el riesgo relativo del indicador abandono de la vacunación.

**Resultados:**

la estadística *scan* espacial detectó la presencia de grupos estadísticamente significativos para el abandono de las cuatro vacunas en todos los años analizados. Sin embargo, el mayor número de grupos con estimaciones altas de riesgos relativos se identificó en 2020. Se destacan las macrorregiones del Vale do Aço y Oeste; Norte y Oeste; y Sudeste para las vacunas pentavalente, antipoliomielítica y contra el rotavirus, respectivamente.

**Conclusión:**

mientras se intentaba disminuir el impacto devastador de la pandemia de COVID-19, retrocedió el programa de inmunización. La presencia de grupos indica que es necesario implementar estrategias integradas que puedan involucrar a diferentes sectores para la búsqueda activa de niños y evitar brotes de enfermedades inmunoprevenibles en el futuro próximo.

**Highlights**

(1) Spatial heterogeneities in vaccination abandonment in the state.

(2) Target groups that need priority interventions.

(3) Revitalizing the immunization program to face the impact generated by COVID-19.

(4) Investing in the production of timely records of the immunization information systems.

## Introduction

SARS-CoV-2, the virus responsible for COVID-19, rapidly evolved from a punctual outbreak in December 2019 in the province of Hubei, China, to a pandemic responsible for more than 200 million confirmed cases and 5 million deaths worldwide by December 2021^1^.

Since then, the public health response measures to mitigate the pandemic have concentrated on social distancing and on quarantine policies, among others^2^. However, these strategies exerted some negative effects. According to a report by the World Health Organization (WHO) published in August 2020, 90% of 105 countries reported at least one interruption in the essential health services, with routine vaccination mentioned among those most frequently affected. The most significant interruptions were reported in low- and middle-income countries^3^.

This situation represents a severe threat to public health that can result in outbreaks of vaccine-preventable diseases, especially among children^4^. The WHO estimates that at least 80 million children will be susceptible to diseases such as measles and poliomyelitis due to the vaccination decline during the COVID-19 pandemic^5^.

In Brazil, the National Surveillance System of the National Immunization Program (*Programa Nacional de Imunizações*, PNI) had already recorded a significant decline in vaccination before the pandemic, with considerable heterogeneities across the municipalities^6^. In addition to the low coverage levels already recorded in the country^6^, another indicator signals one further problem. In 2019, the Brazilian states showed a vaccination abandonment percentage ≥ 10%, a value that is considered high^7^.

The vaccination abandonment percentage is a measure of the strength of the health services and is used in the vaccines with multidose regimes. This indicator evaluates the difference between the number of first doses and the number of last doses administered of the vaccination schedule^8^ since, to be considered properly vaccinated, an individual needs to complete the recommended schedule for each age group or life cycle^9^. 

In this sense, studies should not only seek an analysis of vaccination coverage, which has even been consistently documented^6^ ^,^ ^10^. Research studies on vaccination abandonment should be encouraged since, although vaccination coverage is increasing globally, many children in developing countries still abandon their vaccination schemes^11^.

Given this scenario, singular attention and strategic planning consistent with the characteristics of each location are necessary to reduce vaccination abandonment. One of the methods that can meet this requirement is the spatial scan analysis technique, which has its applicability in public health still restricted to evaluating vaccination abandonment at subnational or regional levels. In addition to that, although recent analyses show the interruptions in routine immunization programs in 2020, especially during the initial phases of the COVID-19 pandemic^12^ ^-^ ^13^, in a systematic literature review no studies were identified that considered the territory in spatial units with a higher disaggregation level, such as Minas Gerais, the second most populous state in Brazil^14^. Consequently, the objective of the current study was to identify spatial clusters of abandonment regarding routine vaccines in children.

## Method

### Type of study

This is an ecological and population-based study.

### Study locus

**Figure 1:**
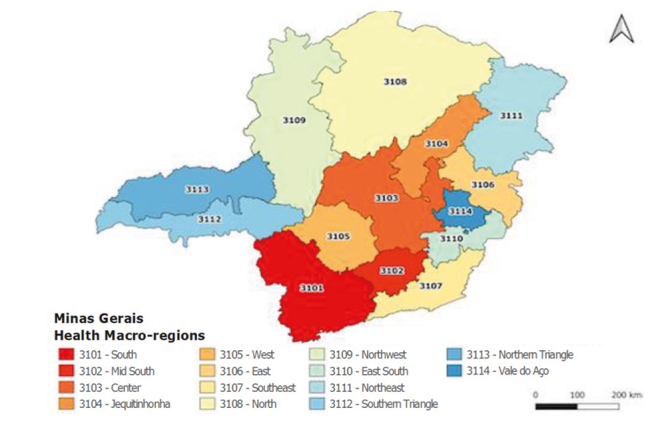
Macro-regions of the state of Minas Gerais, MG, Brazil, 2022

The study was conducted in the state of Minas Gerais, Brazil. For management and planning aspects, the state is divided into fourteen macro-regions: South (3101), Mid South (3102), Center (3103), Jequitinhonha (3104), West (3105), East (3106), Southeast (3107), North (3108), Northwest (3109), East South (3110), Northeast (3111), Southern Triangle (3112), Northern Triangle (3113) and Vale do Aço (3114) (Figure 1). In turn, these macro-regions include 853 municipalities^14^, considered territorial units of analysis for the current study.

### Study population

The population consisted of children aged less than one year old. According to registration in the Live Birth Information System (*Sistema de Informação sobre Nascidos Vivos*, SINASC), totals of 260,959, 263,640 and 256,890 children were born in 2017, 2018 and 2019, respectively, in the state of Minas Gerais^15^, a fraction corresponding to the denominator that makes up the calculation basis of the vaccination abandonment indicator. The data were obtained in April 2022 by accessing the electronic platform of the Unified Health System Informatics Department (DATASUS)^15^.

### Study variables and period

The vaccination abandonment indicators corresponding to poliomyelitis (dose 1: two months old; dose 3: six months old), pentavalent (dose 1: two months old; dose 3: six months old), pneumococcal 10-valent (dose 1: two months old; dose 2: four months old) and human rotavirus oral vaccine (HROV) (dose 1: two months old; dose 2: four months old) were analyzed from January to December of 2018, 2019 and 2020. The number of doses applied was obtained from the Immunization Program Evaluation System (*Sistema de Avaliação do Programa de Imunizações*, SAPI), extracted from the DATASUS in April 2022^16^.

### Data treatment and analysis

In a first stage, the data were stored in Microsoft Excel (2016), where it was possible to calculate the vaccination abandonment indicator. This indicator applies to vaccines with a multi-dose schedule and is calculated from the difference between the number of first and last doses (people who initiated but did not finish the schedule). Subsequently, data consistency was verified. 

To verify the existence of clusters from the vaccination abandonment indicator, the SaTScan 9.6 software was used, supported by Poisson’s discrete model^17^, as the indicator consists of a count and the population exposed to the risk varies according to the municipality, that is, the expected number of abandonment cases is proportional to the size of its population. 

The scan statistics acts by scanning various search radii, reason why it was necessary to define this limit. The 50% radium of the population exposed was stipulated as the spatial detection maximum parameter. Each cluster was statistically tested by the log likelihood ratio test, and the maximum likelihood window was considered as the most likely cluster. Statistical significance was assessed using the Monte Carlo hypothesis tests^18^. 

Finally, relative risk estimates were calculated. This measure allows comparing diverse information from different areas, standardizing it and removing the effect of the populations. With a geographical region formed by clusters denoted as A1, A2, A3..., Ak and with X as a variable that indicates vaccination abandonment, so that each Xi occurrence (i = 1, 2, 3..., k) is associated with the cluster, with population ni (i = 1, 2, 3..., k), the relative risk of a given Ai cluster is the quotient between the vaccination abandonment observed in cluster Ai and vaccination abandonment in the other study regions^19^. 

### Ethical aspects

The study uses data from the unrestricted access public domain, for which there is no identification of the individuals participating in the research; therefore, it waives review by any Research Ethics Committee (*Comitê de Ética em Pesquisa*, CEP).

## Results

**Figure 2:**
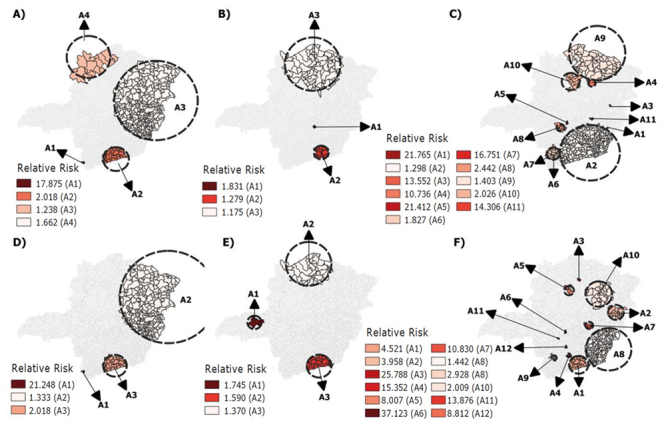
Spatial risk clusters for abandonment of the pentavalent (A: 2018; B: 2019; C: 2020) and poliomyelitis (D: 2018; E: 2019; F: 2020) vaccines in children aged less than one year old (n=781,489). Minas Gerais, MG, Brazil, 2018-2020

Source: Immunization Program Evaluation System (SAPI)/Unified Health System Informatics Department (DATASUS); *A = Risk cluster

**Figure 3:**
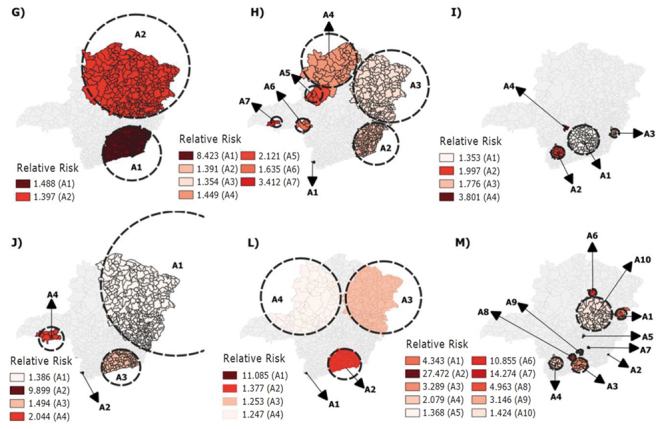
Spatial risk clusters for abandonment of the pneumococcal 10 (G: 2018; H: 2019; I: 2020) and rotavirus (J: 2018; L: 2019; M: 2020) vaccines in children aged less than one year old (n=781,489). Minas Gerais, MG, Brazil, 2022

Source: Immunization Program Evaluation System (SAPI)/Unified Health System Informatics Department (DATASUS); *A = Risk cluster

Between 2018 and 2020, a total of 444,982 (24.63%) vaccine schedules started were abandoned for the pneumococcal 10, poliomyelitis, pentavalent and rotavirus vaccines in the state of Minas Gerais. The spatial scan statistics detected the presence of statistically significant clusters for abandonment of these four vaccines in all the years analyzed (Figures 2 and 3). However, the largest number of clusters with high relative risk estimates was identified in 2020, with the exception of the pneumococcal 10 vaccine. The macro-regions of Vale do Aço (3114) and West (3105); North (3108) and West (3105); and Southeast (3107) stand out for the pentavalent, poliomyelitis and rotavirus vaccines, respectively, in 2020 (Figures 4 and 5). 

Another fact that draws the attention is the absence of clusters in the West macro-region (3105) in 2020 for the rotavirus vaccine, as high relative risks were identified in this macro-region for the other vaccines analyzed (Figures 2 and 3).

**Figure 4:** Characteristics of the significant clusters identified in the scanning analysis for the risk of abandonment corresponding to the pentavalent and poliomyelitis vaccines in children aged less than one year old (n=781,489). Minas Gerais, MG, Brazil, 2018-2020

Vaccine	Year	Risk cluster (A)*	Relative Risk	p-value	No. of municipalities	Macro-region
**Pentavalent**	2018	1	17.875	<0.01	1	3101
		2	2.018	<0.01	41	3101; 3107; 3102
		3	1.238	<0.01	300	3110; 3114; 3106; 3111; 3104; 3103; 3108; 3107
		4	1.662	<0.01	14	3109; 3108
	2019	1	1.831	<0.01	1	3103
		2	1.279	<0.01	21	3102; 3107
		3	1.175	<0.01	71	3109; 3108
	2020	1	21.765	<0.01	1	3114
		2	1.298	<0.01	165	3101; 3102; 3103; 3105; 3107; 3110
		3	13.552	<0.01	1	3106
		4	10.736	<0.01	2	3108
		5	21.412	<0.01	1	3105
		6	1.827	<0.01	24	3101
		7	16.751	<0.01	1	3101
		8	2.442	<0.01	5	3105
		9	1.403	<0.01	56	3108; 3111
		10	2.026	<0.01	11	3108
		11	14.306	<0.01	1	3103
**Poliomyelitis**	2018	1	21.248	<0.01	1	3101
		2	1.333	<0.01	235	3114; 3106; 3111; 3104; 3108; 3103; 3110
		3	2.018	<0.01	40	3101; 3107; 3102
	2019	1	1.745	<0.01	2	3113
		2	1.590	<0.01	40	3101; 3107; 3102
		3	1.370	<0.01	35	3108; 3109
	2020	1	4.521	<0.01	26	3101; 3102; 3107
		2	3.958	<0.01	19	3106; 3111
		3	25.788	<0.01	1	3108
		4	15.352	<0.01	2	3101
		5	8.007	<0.01	4	3108
		6	37.123	<0.01	1	3105
		7	10.830	<0.01	6	3103
		8	1.442	<0.01	163	3102; 3103; 3107; 3110; 3114
		9	2.928	<0.01	3	3101
		10	2.009	<0.01	29	3104; 3108; 3111
		11	13.876	<0.01	1	3105
		12	8.812	<0.01	1	3105

Source: Immunization Program Evaluation System (SAPI)/Unified Health System Informatics Department (DATASUS); *A = Risk cluster

**Figure 5:** Characteristics of the significant clusters identified in the scanning analysis for the risk of abandonment corresponding to the pneumococcal 10 and rotavirus vaccines in children aged less than one year old (n=781,489). Minas Gerais, MG, Brazil, 2018-2020

Vaccine	Year	Risk cluster (A)*	Relative Risk	p-value	No. of municipalities	Macro-region
**Pneumococcal 10**	2018	1	1.488	<0.01	221	3110; 3101; 3107; 3102; 3103; 3105
		2	1.397	<0.01	265	3114; 3106; 3111; 3104; 3109; 3103; 3108
	2019	1	8.423	<0.01	1	3101
		2	1.391	<0.01	158	3110; 3107; 3102; 3114; 3103
		3	1.354	<0.01	188	3114; 3106; 3111; 3104; 3108; 3103; 3110
		4	1.449	<0.01	49	3109; 3108
		5	2.121	<0.01	5	3109
		6	1.635	<0.01	5	3112; 3101
		7	3.412	<0.01	2	3112
	2020	1	1.353	<0.01	114	3101; 3102; 3103; 3105; 3107; 3110
		2	1.997	<0.01	110	3101
		3	1.776	<0.01	13	3107; 3110
		4	3.801	<0.01	1	3105
**Rotavirus**	2018	1	1.386	<0.01	359	3110; 3114; 3106; 3111; 3104; 3103; 3108; 3107
		2	9.899	<0.01	1	3101
		3	1.494	<0.01	112	3101; 3107; 3102
		4	2.044	<0.01	11	3112
	2019	1	11.085	<0.01	1	3101
		2	1.377	<0.01	101	3101;3102; 3107
		3	1.253	<0.01	188	3114; 3106; 3111; 3104; 3108; 3103; 3110
		4	1.247	<0.01	91	3113; 3103; 3109; 3112; 3108; 3105
	2020	1	4.343	<0.01	12	3106; 3111
		2	27.472	<0.01	1	3107
		3	3.289	<0.01	24	3101; 3102; 3107
		4	2.079	<0.01	22	3101
		5	1.368	<0.01	1	3103
		6	10.855	<0.01	2	3108
		7	14.274	<0.01	1	3102
		8	4.963	<0.01	6	3101; 3102
		9	3.146	<0.01	6	3102
		10	1.420	<0.01	77	3103; 3104; 3106; 3108; 3111; 3114

Source: Immunization Program Evaluation System (SAPI)/Unified Health System Informatics Department (DATASUS); *A = Risk cluster

## Discussion

The results of this analysis revealed clusters with risk of abandonment for all the vaccines in Minas Gerais. These findings show the potential of spatial analysis, as target groups that need priority interventions were identified. In addition to that, macro-regions with high relative risks verify that, even in countries with well-established health systems and effective immunization programs such as Brazil, the advances achieved in previous years can be easily lost^6^.

Furthermore, in this study it was possible to elucidate the change in the distribution pattern of the clusters throughout the three years analyzed. The literature shows that difficulties accessing health services^20^, social vulnerability^21^, limited family support^22^, the ideological currents that oppose vaccination^23^ and shortage of vaccines^24^, among other factors, may justify this scenario. However, the 2018 and 2019 data draw the attention, where lower relative risks were identified, mainly close to a value of 1, which can lead to low discrimination power. On the other hand, a large number of clusters with high relative risk estimates was identified in 2020. 

It is likely that the COVID-19 pandemic has intensified the vaccination abandonment scenario. Some structuring elements that may have determined this process are the following: social distancing^25^, strangulation of the health services^26^, lack of human resources and physical and mental exhaustion of the professionals^27^, in addition to a political agenda that came in opposition to the collective protection measures, extending the deleterious effects of the pandemic^28^.

According to the United Nations Children’s Fund (UNICEF)^29^, 23 million children did not receive basic vaccines in 2020, 3.7 million more than in 2019. The national data show a reduction in routine children’s vaccination in March/April 2020 (when the restrictions were higher) when compared to previous years; dose three for the pentavalent and poliomyelitis vaccines administered at six months of age decreased by 18%^30^. Another impacting indicator refers to the reduction in the orders for routine vaccines by the national or regional authorities, when compared to the 2019 standards^31^.

Brazil was severely affected by COVID-19, with rapid spatial dissemination of cases and deaths. At the end of May 2020, Latin America was declared as the epicenter of the COVID-19 pandemic, mainly because of Brazil. However, the epidemiological curves in the country conceal different patterns for notifying the disease in the different administrative units^32^. In Minas Gerais, the curve of COVID-19 infection cases was increased at the end of April 2020^33^. In fact, it was during this month that all macro-regions of the state presented the highest isolation rate between March and November 2020 (above 40%)^34^. 

Although noticing certain relaxation in social distancing throughout the subsequent months, the overall mean in all macro-regions of the state was above 35%^34^. Therefore, it is possible that the children had initiated the vaccination schedule before the measures implemented to mitigate COVID-19 transmission, but did not conclude it.

Although social distancing has been recommended, a previous epidemic shows that substantial vaccination gaps increase the risk of vaccine-preventable disease outbreaks as pre-pandemic social contact is resumed^35^. A recent study has shown that the deaths that are preventable with routine vaccination outweigh the excess risk of death due to COVID-19 associated with attending a health service for vaccination^36^. 

Although the 2020 data pointed to a clear increase in the risk of abandoning the vaccination schedule, in this study, a cluster with a high relative risk (8.423) for the pneumococcal 10 vaccine was observed in 2019 in the South macro-region. This is not an isolated phenomenon, as another cluster was also identified for the rotavirus vaccine in this same macro-region in 2019. According to the vaccination calendar, the second dose of the pneumococcal 10 and rotavirus vaccines must be administered at the age of four months old^37^. It is likely that the low demand by the parents/guardians^38^, as well as the insufficient performance of health surveillance actions, such as guidance and active search during home visits by health professionals, have contributed to this result^39^.

Another detail that draws the attention is the fact that the clusters for the different vaccines do not coincide, as the first and second doses of all the vaccines analyzed are applied at two and four months of age, respectively; and the third dose of the pentavalent and poliomyelitis vaccines are administered at six months old^37^. In a systematic review, it was found that multiple injections per visit to the health unit can lead to vaccination abandonment, attributed to the parents’ concern with pain and suffering in their children^40^. However, this justification is not applicable to the rotavirus vaccine, as it is administered through the oral route^37^. 

Non-concomitancy in application of the vaccines can also be associated with the professional performance since, although the PNI has systematically invested in training sessions^41^, there is turnover of health professionals, among them those who work in vaccination rooms^42^. The schedule became more complex, demanding greater knowledge from the professionals about the vaccination regimes and their updates, especially for children who arrive at the units with delayed vaccines^41^. 

However, it is important to mention that compliance with the vaccination schedule should not be exclusively linked to the children’s visits to the services, but also to the periodic home visits by health professionals. A study conducted in the Democratic Republic of Congo showed that one of the predictors for vaccination abandonment among children was lack of a reminder system in the days prior to the scheduled vaccination^43^.

Another element that should be mentioned is the absence of clusters in the West macro-region (3105) in 2020 for the rotavirus vaccine, as high relative risks were identified in this region for the other vaccines analyzed. It is possible that the administration route explains this result. Administration through the oral route is preferable to the traditional injection-based formulations^44^. The possible effect of the quality of the data presented in the Brazilian information system is added to that discussion. Despite the benefits and being in an advanced implementation phase, the scarcity of trained human resources, the deficit in information technology and the ineffectiveness of the constant updating of Health Information Systems are challenges for the production of timely records^45^. Such situation is even more worrying in regions where the significant demand for services is higher due to the large population contingent, such as the state of Minas Gerais^45^. That situation has already been reported in the international scenario as well. In Ghana, a study attributed the values found for the “vaccination abandonment” indicator to deficient data management^46^.

Another issue worth highlighting is vaccination associated with the socioeconomic conditions^47^. In Minas Gerais, the Vale do Aço, North and Southwest macro-regions, with high relative risks for vaccination abandonment, fall into the Human Development Index (HDI) average range^48^. A research study conducted in 76 countries showed that a high HDI is a predictor for greater sensitization and regulation of the vaccination actions^49^. Paradoxically, there is growing evidence that vaccine incompleteness and hesitation occur among the higher-income population strata^50^ ^-^ ^51^. In this study, the West macro-region, with the fourth best HDI in the state (classified as high)^48^, also presented a high relative risk for vaccination abandonment. 

Therefore, for future studies, epidemiological household surveys would be appropriate, particularly in the clusters identified in this research, in order to elucidate gaps that permeate the administrative estimates; in addition to that, developing research studies that explore the facilitating and hindering elements in data recording, for example, through participant observation.

Finally, as shown by the COVID-19 pandemic, having granular (detailed) data is crucial to conduct targeted interventions. Thus, the results of this study confer visibility to the “abandonment of routine vaccines” problem and show the importance for health professionals and managers to implement strategies for an active search of children in an equitable way.

Among the limitations of this study it is worth noting the data source employed. DATASUS was used in this research. In it, registration of the vaccines applied is performed offline, which requires that those responsible in each municipality send all the information. Thus, the data between the local level and the numbers consolidated at the national level can be different. However, despite this, choice of this type of source reduces the operating costs and does not preclude analyses. In addition, identification of the target population, for which the SINASC is used as a basis, can also contain inaccuracies due to errors in population estimates, migration flows and population mobility^52^. Nevertheless, these limitations do not minimize the potential that this system represents both for management and for research studies.

## Conclusion

The paper raises a reflection on the possible impact of the COVID-19 pandemic on abandonment of the routine vaccination schedule in children aged less than one year old in the state of Minas Gerais, given the presence of spatial clusters with high relative risks in 2020 when compared to previous years. In the meantime, it is urgent to take a close look at the clusters, with a view to preventing resurgence/worsening of vaccine-preventable diseases. In addition to that, vaccination records and data quality are issues requiring attention, as the results are influenced by data imprecisions. 

## References

[1] World Health Organization (2021). WHO Coronavirus (Covid-19) Dashboard. Situation by Region, Country,
Territory & Area.

[2] Nussbaumer-Streit B, Mayr V, Dobrescu AI, Chapman A, Persad E, Klerings I (2020). Quarantine alone or in combination with other public health
measures to control COVID-19: a rapid review. Cochrane Database Syst Rev.

[3] World Health Organization (2020). Pulse survey on continuity of essential health services during the
COVID-19 pandemic. Interim report.

[4] Patel Murthy B, Zell E, Kirtland K, Jones-Jack N, Harris L, Sprague C (2021). Impact of the COVID-19 Pandemic on Administration of Selected
Routine Childhood and Adolescent Vaccinations - 10 U S. Jurisdictions,
March-September 2020. MMWR Morb Mortal Wkly Rep.

[5] World Health Organization (2020). At least 80 million children under one at risk of diseases such as
diphtheria, measles and polio as COVID-19 disrupts routine vaccination
efforts, warn Gavi, WHO and UNICEF.

[6] Arroyo LH, Ramos ACV, Yamamura M, Weiller TH, Crispim JA, Cartagena-Ramos D (2020). Areas with declining vaccination coverage for BCG, poliomyelitis,
and MMR in Brazil (2006-2016): maps of regional
heterogeneity. Cad Saude Publica.

[7] World Health Organization (2020). Progress and Challenges with Achieving Universal Immunization Coverage.
2019 WHO/UNICEF Estimates of National Immunization Coverage.

[8] Fonseca KR, Buenafuente SMF (2021). Analysis of vaccination coverage of children under one year old
in Roraima, Brazil, 2013-2017. Epidemiol Serv Saude.

[9] Ministério da Saúde. Secretaria de Vigilância em Saúde. Departamento
de Imunização e Doenças Transmissíveis. Coordenação Geral do Programa
Nacional de Imunizações (2020). Informe técnico. Estratégia de recuperação do esquema de vacinação
atrasado de crianças menores de 5 anos de idade.

[10] Utazi CE, Wagai J, Pannell O, Cutts FT, Rhoda DA, Ferrari MJ (2020). Geospatial variation in measles vaccine coverage through routine
and campaign strategies in Nigeria: Analysis of recent household
surveys. Vaccine.

[11] Chanie MG, Ewunetie GE, Molla A, Muche A (2021). Determinants of vaccination dropout among children 12-23 months
age in north Gondar zone, northwest Ethiopia, 2019. PLoS One.

[12] Lassi ZS, Naseem R, Salam RA, Siddiqui F, Das JK (2021). The Impact of the COVID-19 Pandemic on Immunization Campaigns and
Programs: A Systematic Review. Int J Environ Res Public Health.

[13] Masresha BG, Luce R, Shibeshi ME, Ntsama B, N'Diaye A, Chakauya J (2020). The performance of routine immunization in selected African
countries during the first six months of the COVID-19
pandemic. Pan Afr Med J.

[14] Instituto Brasileiro de Geografia e Estatística (BR) (2021). Cidades e Estados. Minas Gerais.

[15] Ministério da Saúde (BR), Secretaria de Vigilância em Saúde,
Departamento de Análise de Situaçãode Saúde (2021). Nascidos vivos - Brasil.

[16] Ministério da Saúde (BR), Secretaria de Vigilância em Saúde,
Departamento de Vigilância Epidemiológica, Coordenação Geral do Programa
Nacional de Imunizações (2021). Imunizações - doses aplicadas.

[17] Kulldorff M (1997). A spatial scan statistic. Commun Stat - Theory Methods.

[18] Fay MP, Follmann DA (2002). Designing Monte Carlo Implementations of Permutation or Bootstrap
Hypothesis Tests. Am Stat.

[19] Rodrigues RN, Leano HAM, Bueno IC, Araújo KMFA, Lana FCF (2020). High-risk areas of leprosy in Brazil between
2001-2015. Rev Bras Enferm.

[20] Duarte DC, Oliveira VC, Guimarães EAA, Viegas SMF (2019). Vaccination access in Primary Care from the user's perspective:
senses and feelings about healthcare services. Esc Anna Nery.

[21] Song IH, Palley E, Atteraya MS (2020). Inequalities in complete childhood immunisation in Nepal: results
from a population-based cross-sectional study. BMJ Open.

[22] Powelson J, Magadzire BP, Draiva A, Denno D, Ibraimo A, Benate BBL (2022). Determinants of immunisation dropout among children under the age
of 2 in Zambézia province, Mozambique: a community-based participatory
research study using Photovoice. BMJ Open.

[23] Baumgaertner B, Carlisle JE, Justwan F (2018). The influence of political ideology and trust on willingness to
vaccinate. PLoS One.

[24] Ministério da Saúde (MS), Secretaria de Vigilância em Saúde,
Coordenação Geral do Programa Nacional de Imunizações (2017). Nota Informativa nº17 - Coordenação-Geral do Programa Nacional de
Imunizações.

[25] McDonald HI, Tessier E, White JM, Woodruff M, Knowles C, Bates C (2020). Early impact of the coronavirus disease (COVID-19) pandemic and
physical distancing measures on routine childhood vaccinations in England,
January to April 2020. Euro Surveill.

[26] Oliveira WK, Duarte E, França GVA, Garcia LP (2020). How Brazil can hold back COVID-19. Epidemiol Serv Saude.

[27] Morgantini LA, Naha U, Wang H, Francavilla S, Acar Ö, Flores JM (2020). Factors contributing to healthcare professional burnout during
the COVID-19 pandemic: A rapid turnaround global survey. PLoS One.

[28] Pereira AK, Oliveira MS, Sampaio TS (2020). Heterogeneidades das políticas estaduais de distanciamento social
diante da COVID-19: aspectos políticos e técnicos
administrativos. Rev Admin Publica.

[29] United Nations Children's Fund (2021). COVID-19 pandemic leads to major backsliding on childhood vaccinations,
new WHO, UNICEF data shows.

[30] Silveira MF, Tonial CT, Maranhão AGK, Teixeira AMS, Hallal PC, Menezes AMB (2021). Missed childhood immunizations during the COVID-19 pandemic in
Brazil: Analyses of routine statistics and of a national household
survey. Vaccine.

[31] Santoli JM, Lindley MC, DeSilva MB, Kharbanda EO, Daley MF, Galloway L (2020). Effects of the COVID-19 Pandemic on Routine Pediatric Vaccine
Ordering and Administration - United States, 2020. MMWR Morb Mortal Wkly Rep.

[32] Castro MC, Kim S, Barberia L, Ribeiro AF, Gurzenda S, Ribeiro KB (2021). Spatiotemporal pattern of COVID-19 spread in
Brazil. Science.

[33] Amaral P, Andrade LM, Fonseca FG, Perez J (2021). Impact of COVID-19 in Minas Gerais, Brazil: Excess deaths,
sub-notified cases, geographic and ethnic distribution. Transbound Emerg Dis.

[34] Minas Gerais (Estado), Secretaria do Estado de Saúde (2021). Boletim Epidemiológico e Assistencial COVID-19 (Edição Especial):
Avaliação do Isolamento.

[35] Nagbe T, Williams GS, Rude JM, Flomo S, Yeabah T, Fallah M (2019). Lessons learned from detecting and responding to recurrent
measles outbreak in Liberia post Ebola-Epidemic 2016-2017. Pan Afr Med J.

[36] Abbas K, Procter SR, Van Zandvoort K, Clark A, Funk S, Mengistu T (2020). LSHTM CMMID COVID-19 Working Group. Routine childhood
immunisation during the COVID-19 pandemic in Africa: a benefit-risk analysis
of health benefits versus excess risk of SARS-CoV-2
infection. Lancet Glob Health.

[37] Ministério da Saúde (BR) (2020). Calendário da Criança.

[38] Hadjipanayis A, van Esso D, Del Torso S, Dornbusch HJ, Michailidou K, Minicuci N (2020). Vaccine confidence among parents: Large scale study in eighteen
European countries. Vaccine.

[39] Lemos PL, Oliveira GJ, Souza NFC, Silva IM, Paula IPG, Silva KC (2021). Factors associated with the incomplete opportune vaccination
schedule up to 12 months of age, Rondonópolis, Mato Grosso. Rev Paul Pediatr.

[40] Wallace AS, Mantel C, Mayers G, Mansoor O, Gindler JS, Hyde TB (2014). Experiences with provider and parental attitudes and practices
regarding the administration of multiple injections during infant
vaccination visits: lessons for vaccine introduction. Vaccine.

[41] Domingues CMAS, Maranhão AGK, Teixeira AM, Fantinato FFS, Domingues RAS (2020). The Brazilian National Immunization Program 46 years of
achievements and challenges. Cad Saude Publica.

[42] Martins JRT, Viegas SMF, Oliveira VC, Rennó HMS (2019). Vaccination in everyday life: experiences indicate Permanent
Education. Esc Anna Nery.

[43] Kayembe-Ntumba HC, Vangola F, Ansobi P, Kapour G, Bokabo E, Mandja BE (2022). Vaccination dropout rates among children aged 12-23 months in
Democratic Republic of the Congo: a cross-sectional study. Arch Public Health.

[44] Vela Ramirez JE, Sharpe LA, Peppas NA (2017). Current state and challenges in developing oral
vaccines. Adv Drug Deliv Rev.

[45] Silva BS, Souza KC, Souza RG, Rodrigues SB, Oliveira VC, Guimarães EAA (2020). Structural and procedural conditions in National Immunization
Program Information System establishment. Rev Bras Enferm.

[46] Baguune B, Ndago JA, Adokiya MN (2017). Immunization dropout rate and data quality among children 12-23
months of age in Ghana. Arch Public Health.

[47] Allan S, Adetifa IMO, Abbas K (2021). Inequities in childhood immunisation coverage associated with
socioeconomic, geographic, maternal, child, and place of birth
characteristics in Kenya. BMC Infect Dis.

[48] Minas Gerais (Estado). Secretaria de Estado de Planejamento e
Gestão (2015). Plano Mineiro de Desenvolvimento Integrado PMDI 2016 - 2027 Perfis
Territoriais.

[49] García-Toledano E, Palomares-Ruiz A, Cebrián-Martínez A, López-Parra E (2021). Health Education and Vaccination for the Construction of
Inclusive Societies. Vaccines.

[50] Buffarini R, Barros FC, Silveira MF (2020). Vaccine coverage within the first year of life and associated
factors with incomplete immunization in a Brazilian birth
cohort. Arch Public Health.

[51] Silveira MF, Buffarini R, Bertoldi AD, Santos IS, Barros AJD, Matijasevich A (2020). The emergence of vaccine hesitancy among upper-class Brazilians:
Results from four birth cohorts, 1982-2015. Vaccine.

[52] Domingues CMAS, Teixeira AMS (2013). Vaccination coverage and impact on vaccine-preventable diseases
in Brazil between 1982 and 2012: National Immunization Program progress and
challenges. Epidemiol Serv Saude.

